# CDK4/6 inhibitors in breast cancer therapy: mechanisms of drug resistance and strategies for treatment

**DOI:** 10.3389/fphar.2025.1549520

**Published:** 2025-05-12

**Authors:** Tong Gao, Ying Sun, Ping Leng, Donghua Liu, Qie Guo, Jing Li

**Affiliations:** ^1^ Department of Pharmacy, The Affiliated Hospital of Qingdao University, Qingdao, China; ^2^ Department of Health Management Center, The Affiliated Hospital of Qingdao University, Qingdao, China

**Keywords:** CDK4/6 inhibitors, cell cycle arrest, immune regulation, acquired resistance, combination therapy

## Abstract

Dysregulated cell cycle progression is a well-established hallmark of cancer, driving the development of targeted antitumor therapies that intervene at specific phases of the cell cycle. Among these therapeutic targets, cyclin-dependent kinases 4 and 6 (CDK4/6) have emerged as critical regulators of cell cycle progression, with their aberrant activation being strongly implicated in tumorigenesis and cancer progression. Currently, multiple CDK4/6 inhibitors have received clinical approval for hormone receptor (HR)-positive/human epidermal growth factor receptor 2 (HER2)-negative breast cancer, demonstrating dual therapeutic mechanisms through both cell cycle arrest and enhancement of antitumor immunity. However, clinical implementation faces two major challenges: the inevitable development of acquired resistance during prolonged treatment, and the need for optimized combination strategies with other anticancer agents to achieve synergistic efficacy. This review systematically examines the molecular mechanisms underlying CDK4/6 inhibitor function and characterizes currently approved therapeutic agents. Importantly, it synthesizes recent discoveries regarding resistance mechanisms, including dysregulated cell cycle checkpoints, compensatory signaling pathway activation, and tumor microenvironment adaptations. Furthermore, we critically evaluate emerging combination therapeutic approaches targeting these resistance mechanisms. By integrating mechanistic insights with clinical evidence, this analysis aims to provide actionable strategies for overcoming therapeutic resistance and maximizing the clinical potential of CDK4/6 inhibitors in breast cancer management.

## 1 Introduction

The cell cycle encompasses the series of events through which actively dividing cells progress from the completion of one division to the next, sequentially traversing the G1, S, G2, and M phases (Matthews et al., 2022). This process is governed by tightly regulated checkpoints that enforce strict quality control, ensuring genomic fidelity before progression to subsequent phases. In cancer, however, dysregulation of cell cycle control mechanisms leads to uncontrolled proliferation—a hallmark of malignancy. Targeting cell cycle regulators has consequently emerged as a cornerstone of modern oncology drug development (Asghar et al., 2015). Cyclin dependent kinases (CDKs) are key kinases involved in cell cycle regulation, which play an important role in cell cycle initiation and transition regulation at various stages (Jirawatnotai et al., 2020). There are four types of CDK involved in the regulation of cell growth cycle in human body, namely CDK1/2/4/6. While CDK1 and CDK2 regulate mitotic entry (M-phase) and G1/S transition respectively. CDK4/6 specifically drive G1-phase progression by phosphorylating retinoblastoma (Rb) protein to promote cell cycle commitment, which have gained prominence as therapeutic targets (Guo et al., 2025; Rubin et al., 2020). Notably, all clinically approved CDK inhibitors to date target CDK4/6, reflecting their pivotal role in oncogenic proliferation (Goel et al., 2022; Fassl et al., 2022). These inhibitors induce G1-phase arrest by blocking Rb phosphorylation, thereby suppressing tumor growth across multiple malignancies. Their clinical impact, is most pronounced in hormone receptor-positive (HR+) and human epidermal growth factor receptor 2-negative (HER2-) breast cancer, which accounts for approximately 70% of all breast malignancies (Parikh et al., 2024; Jia et al., 2025).

The preferential efficacy of CDK4/6 inhibitors in HR+/HER2- breast cancer stems from this subtype’s unique molecular dependencies. HR+ tumors rely on estrogen receptor (ER)-mediated cyclin D1 upregulation to activate CDK4/6, creating therapeutic vulnerability. HER2- status further preserves this dependency, as HER2 signaling can bypass CDK4/6 through alternative pathways (Parikh et al., 2024; Jia et al., 2025). Over 80% of metastatic HR+/HER2− breast cancer patients now receive CDK4/6 inhibitors as first-line therapy (Goel et al., 2022; Fassl et al., 2022; Parikh et al., 2024; Jia et al., 2025). Despite this success, acquired resistance develops in >50% of patients within 2 years of treatment initiation, often mediated by Rb loss, cyclin E amplification, or PI3K/AKT/mTOR pathway activation. Understanding these mechanisms is critical given that resistance-driven progression accounts for >90% of deaths in metastatic HR+/HER2− breast cancer (Álvarez-Fernández and Malumbres, 2020). This review systematically examines CDK4/6 inhibitor pharmacology, delineates resistance pathways, and evaluates emerging strategies to overcome therapeutic limitations.

## 2 Mechanism of CDK4/6 inhibitors

CDK4/6 inhibitor is a class of drugs with cell cycle regulation function, and their mechanism of action is mainly to regulate the activity of CDK4 and CDK6 subtypes. The primary function of CDK4/6 inhibitors is to block the cell cycle and thus control the cell proliferation process. Recent studies have shown that CDK4/6 inhibitors also play an important role in inducing antitumor immunity (Klein et al., 2018).

### 2.1 Blocking the tumor cell cycle

**FIGURE 1:**
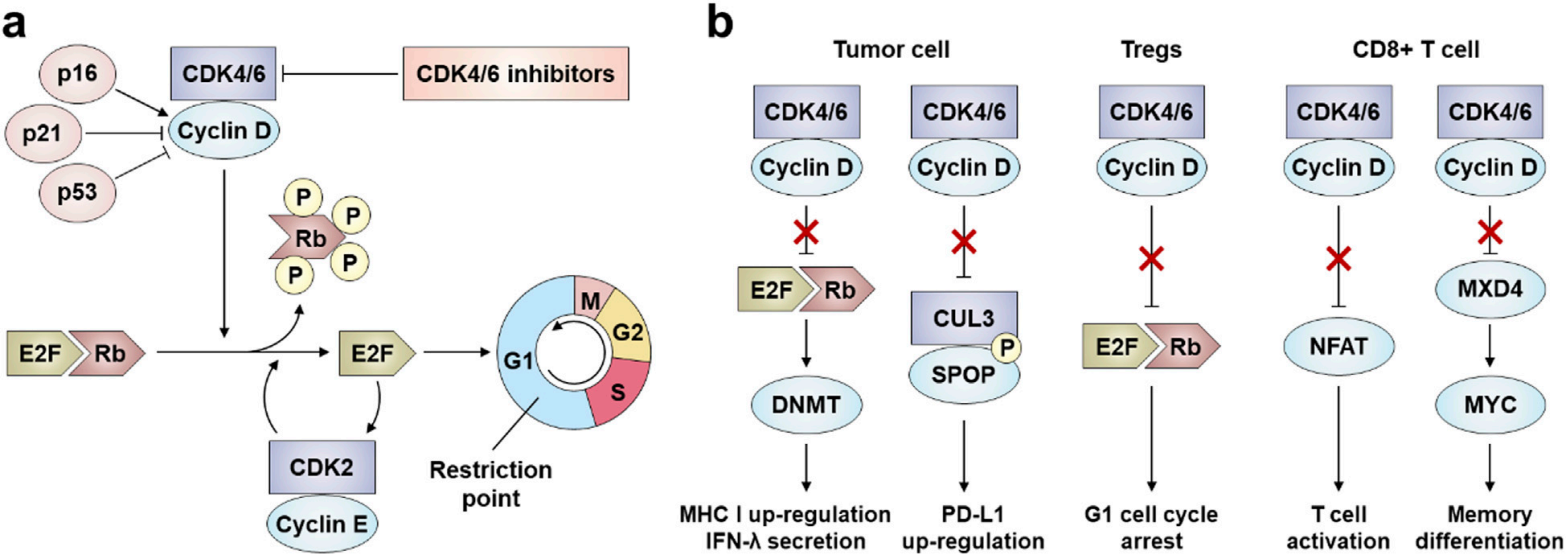
Activation mechanism of CDK4/6. **(a)** Blocking the tumor cell cycle. **(b)** Enhancing the antitumor immunity.

CDK4 and CDK6 are pivotal regulators of the G1-to-S phase transition. During cell cycle re-entry from quiescence into early G1 phase, rising cyclin D levels promote the formation of CDK4/6-cyclin D heterodimeric complexes, which phosphorylate the retinoblastoma protein (Rb) (Wu et al., 2020). In their unphosphorylated state, Rb family proteins bind and inhibit E2F transcription factors, suppressing S-phase gene expression. Phosphorylation by CDK4/6-cyclin D complexes reduces Rb-E2F binding affinity, enabling E2F-mediated transcriptional activation of cell cycle progression genes and driving G1/S transition (Figure 1a) (Dick et al., 2018). Overactivation of CDK4/6 accelerates the G1/S phase transition, forcing cells to prematurely enter S phase without completing DNA damage repair or replication stress checkpoint responses. This leads to the accumulation of replication errors and DNA breaks. Additionally, CDK4/6 may phosphorylate non-canonical substrates, disrupting mitotic spindle assembly and elevating the risk of Chromosome missegregation (Jirawatnotai et al., 2020; Wu et al., 2020). CDK4/6 activity is further modulated by two inhibitory protein families: the INK4 family (p16, p15, p18, and p19), which competitively blocks cyclin D binding to CDK4/6, and the CIP/KIP family (p21, p27 and p53), which exhibits dual regulatory roles (Sherr and Roberts, 1999; Chen et al., 2025). p16 is the most widely characterized, while p16 stabilizes inactive CDK4/6 conformations to prevent Rb phosphorylation, p27 paradoxically assists CDK4/6-cyclin D assembly but requires proteasomal degradation for full kinase activation (Shapiro et al., 1995; Lee et al., 1995). Notably, persistent p27 binding diminishes CDK4/6 inhibitor efficacy by shielding the kinase complex from pharmacological interference (Guiley et al., 2019).

CDK4/6 overexpression is a hallmark of numerous malignancies, driving tumorigenesis through Rb hyperphosphorylation-induced genomic instability and dysregulated cell cycle progression, which accelerates G1/S phase transition and enables uncontrolled proliferation (Krupa et al., 2025). Oncogenic signaling cascades—including JAK/STAT, PI3K/AKT/mTOR, RAS/RAF/MEK/ERK, BTK/NF-κB, and Wnt/β-catenin pathways—converge on the CDK4/6-cyclin D-Rb axis to sustain malignant growth by bypassing cell cycle checkpoints (Qi and Ouyang, 2022; Schoninger and Blain, 2020; Presti and Quaquarini, 2019). Concurrently, tumor suppressor mutations (e.g., p53 loss) indirectly activate this axis via p21 CIP1 suppression, further entrenching CDK4/6 as a central hub for oncogenic signaling (Engeland, 2022). Preclinical studies demonstrate CDK4/6 knockout selectively inhibits tumor growth while sparing normal cells, underscoring its therapeutic potential (Moreno et al., 2025; Zhang et al., 2025). Clinically approved CDK4/6 inhibitors exploit this specificity by competitively blocking cyclin D binding, thereby preventing Rb phosphorylation and E2F-mediated S-phase gene transcription to restore cell cycle control (Gao et al., 2020). Therapeutic efficacy critically depends on intact CDK4/6-cyclin D-Rb signaling, with heightened sensitivity observed in tumors exhibiting pathway dependency—such as ER-positive breast cancers with cyclin D1 amplification and Rb-proficient colorectal carcinomas (Klein et al., 2018; Spring et al., 2020; Vasaikar et al., 2019).

### 2.2 Enhancing the antitumor immunity

CDK4/6 inhibitors exert dual anticancer effects by directly arresting tumor cell proliferation and enhancing immunogenicity to activate antitumor immunity (Figure 1b) (Klein et al., 2018). Clinical analyses of patient biopsies reveal robust upregulation of inflammatory and immune-related genes following CDK4/6 inhibitor treatment, including components of interferon (IFN) signaling and antigen presentation pathways (André et al., 2023; Goodwin et al., 2023). Mechanistically, these inhibitors induce tumor cell expression of endogenous retroviral elements through genome-wide retrotransposon hypomethylation—a process mediated by E2F-dependent suppression of DNA methyltransferase (DNMT) expression during Rb-mediated cell cycle arrest (Shen et al., 2025; Roulois et al., 2015). The resultant accumulation of double-stranded RNA triggers antiviral defense mechanisms, upregulating major histocompatibility complex (MHC) class I molecules and type III IFN (IFN-λ), which promote cytotoxic T lymphocyte recognition and tumor cell elimination (Kimura et al., 2003). Furthermore, CDK4/6 inhibitors increase PD-L1 surface expression via dual regulation: (1) blocking cyclin D-CDK4-mediated phosphorylation of SPOP, a substrate recognition component of the Cullin 3 E3 ubiquitin ligase complex, thereby stabilizing PD-L1 by impairing its proteasomal degradation (Dougan et al., 2019; Zhang et al., 2018). And (2) enhancing transcriptional activation of PD-L1 through IFN signaling potentiation (Schaer et al., 2018). These immunomodulatory synergies not only amplify intrinsic antitumor responses but also sensitize tumors to immune checkpoint blockade therapies.

Beyond their direct antitumor effects, CDK4/6 inhibitors modulate T-lymphocyte immunity through multifaceted mechanisms. These agents enhance cytotoxic T-cell activation while selectively suppressing regulatory T-cells (Tregs) proliferation, thereby reshaping the intratumoral T-cell repertoire toward effector/memory phenotypes (Ameratunga et al., 2019). The nuclear factor of activated T cell (NFAT) protein family is a class of transcription factors that are critical for T cell activation (Macian, 2005). Mechanistically, CDK4/6 inhibitors potentiate T-cell responses via nuclear factor of activated T cells (NFAT) activation: they induce IL-2 secretion and phosphorylate NFAT to upregulate CXCL9/CXCL10 chemokines, facilitating T-cell infiltration (Zhang et al., 2018; Deng et al., 2018). Tregs exhibit heightened CDK6 dependency, rendering them particularly vulnerable to CDK4/6 inhibition through dual epigenetic and cell cycle regulation—downregulating DNA methyltransferase (DNMT) and activating p21 CIP1, which disproportionately reduces Treg populations compared to CD8^+^ cytotoxic T-cells (Malumbres et al., 2004; Obata et al., 2014). Concurrently, CDK4/6 inhibitors promote immunological memory by skewing CD8^+^ T-cells toward central memory precursors via MYC target gene suppression, thereby sustaining long-term antitumor immunity (Heckler et al., 2021).

## 3 CDK4/6 inhibitors approved in clinical

Currently, five CDK4/6 inhibitors—Palbociclib, Ribociclib, Abemaciclib, Dalpiciclib, and Trilaciclib—have been globally approved for clinical use (Table 1). While Trilaciclib is specifically indicated to mitigate chemotherapy-induced myelosuppression, the remaining four agents are predominantly utilized in combination with endocrine therapies for HR+/HER2- breast cancer, reflecting their established role as first-line treatments in this molecular subtype.

**TABLE 1:** CDK4/6 inhibitors approved in clinical.

Agent, structural and Selectivity (IC_50_)	Clinical development (patient populations)	Clinical trials and efficacy	Side effects
Palbociclib (Ibrance®) 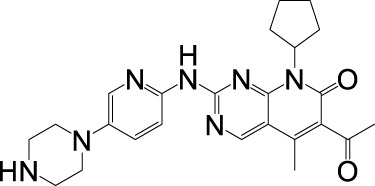 CDK4 (11 nM), CDK6 (16 nM)	• HR-positive and HER2-negative locally advanced or metastatic breast cancer in combination with hormonal therapy	• PALOMA-2 Palbociclib + Letrozole vs. Placebo + Letrozole (median PFS: 38.8 months vs. 28.8 months)	• neutropenia, infection, leukopenia, fatigue, nausea, stomatitis, anemia, alopecia, and diarrhea
Ribociclib (Kisqali®) 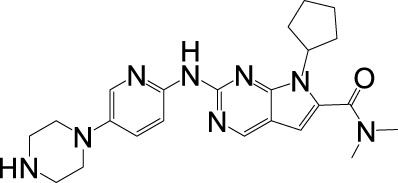 CDK4 (10 nM), CDK6 (39 nM)	• HR-positive and HER2-negative locally advanced or metastatic breast cancer in combination with hormonal therapy	• MONALEESA-2 Ribociclib + Letrozole vs. Placebo + Letrozole (median PFS: 25.3 months vs. 16.0 months) • MONALEESA-7 Ribociclib + Tamoxifen or NSAI + Goserelin vs. Placebo + Tamoxifen or NSAI + Goserelin (median PFS: 23.8 months vs. 13.0 months)	• neutropenia, infection, nausea, fatigue, diarrhea, leukopenia, vomiting, pain, constipation, hair loss, cough, rash, back pain, anemia, and abnormal liver function tests
Abemaciclib (Verzenio®) 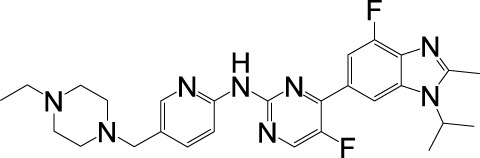 CDK4 (2 nM), CDK6 (10 nM)	• HR-positive and HER2-negative advanced breast cancer in combination with hormonal therapy • Monotherapy for advanced HR-positive and HER2-negative breast cancer • Adjuvant therapy for high-risk, early-stage HR-positive, HER2-negative and lymph node positive breast cancer in combination with hormonal therapy	• MONARCH E Abemaciclib + Endocrine therapy vs. Endocrine therapy (4-year IDFS: 85.8% vs. 79.4%) • MONARCH 3 Abemaciclib + Anastrozole or Letrozole vs. Placebo + Anastrozole or Letrozole (median PFS: 28.18 months vs. 14.76 months) • MONARCH 2 Abemaciclib + Ffulvestrant vs. Placebo + Ffulvestrant (median PFS: 16.4 months vs. 9.3 months)	• diarrhea, infection, neutropenia, leukopenia, anemia, fatigue, nausea, vomiting, hair loss, and decreased appetite
Dalpiciclib (艾瑞康®) 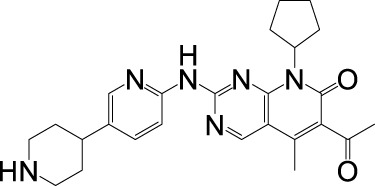 CDK4 (12 nM), CDK6 (10 nM)	• Approved for HR-positive and HER2-negative breast cancer in combination with hormonal therapy	• SHR6390-III-301 Dalpiciclib + Fulvestrant vs. Placebo + Fulvestrant (median PFS: 15.7 months vs. 7.2 months)	• decreased neutrophil counts, decreased white blood cell counts, anemia, decreased platelet counts, rash, elevated liver enzymes, nausea, decreased lymphocyte counts, skeletal muscle pain, oral catarrh, fatigue, and elevated serum creatinine
Trilaciclib (Cosela®) 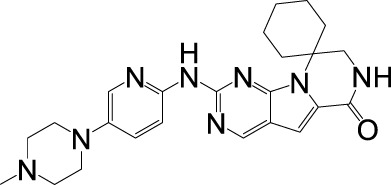 CDK4 (1 nM), CDK6 (4 nM)	• Approved to reduce chemotherapy-induced bone marrow suppression in patients with extensive-stage SCLC.	• GIT28-02 Trilaciclib + Etoposide/Carboplatin vs. Placebo + Etoposide/Carboplatin (patients with severe neutropenia 5.1% vs. 42.1%)	• injection site reactions, acute drug hypersensitivity, and interstitial lung disease/pulmonary inflammation

### 3.1 Palbociclib

Palbociclib, developed by Pfizer as the first oral selective CDK4/6 inhibitor, received FDA approval in February 2015 for HR+HER2- locally advanced or metastatic breast cancer, specifically in combination with aromatase inhibitors as first-line endocrine therapy for postmenopausal women (Samjoo et al., 2024). Administered at 125 mg once daily via a 21-day on/7-day off regimen within a 28-day cycle, treatment continuation is contingent on sustained clinical benefit and tolerable toxicity (Rauthan et al., 2024). Pharmacokinetic studies demonstrate peak plasma concentrations (Cmax) occurring 6–12 h post-dose, with an absolute bioavailability of 46%. Hepatic metabolism predominates via sulfation (SULT2A1-mediated) and oxidation (CYP3A4-mediated) pathways, complemented by secondary glucuronidation and acylation processes, collectively governing its systemic clearance (Groenland et al., 2020).

The PALOMA-2 trial, a randomized double-blind phase III study, evaluated palbociclib plus letrozole versus letrozole alone in 666 postmenopausal women with treatment-naïve, unresectable, locally advanced or metastatic HR+/HER2- breast cancer. Participants were randomized 2:1 to receive palbociclib (125 mg, 21/7-day cycle) + letrozole or placebo + letrozole, with investigator-assessed progression-free survival (PFS) by RECIST v1.1 as the primary endpoint. The palbociclib arm demonstrated superior efficacy, achieving a median PFS of 38.8 months versus 28.8 months in the placebo group, reducing the risk of disease progression and the risk of survival and death (Rugo et al., 2024; McShane et al., 2018). In clinical studies, the most common (≥20%) adverse events of any grade reported in patients treated with Palbociclib were neutropenia, infection, leukopenia, fatigue, nausea, stomatitis, anemia, alopecia, and diarrhea. The most common (≥2%) grade ≥3 adverse events associated with Palbociclib were neutropenia, leukopenia, anemia, fatigue/infection, and elevated aspartate amino acid transferase (Cazzaniga et al., 2019; Beaver et al., 2015).

### 3.2 Ribociclib

Ribociclib, a Novartis-developed oral CDK4/6 inhibitor structurally analogous to palbociclib, received FDA approval in March 2017 as the first-in-class agent for premenopausal women with HR+HER2- locally advanced or metastatic breast cancer. It is indicated in combination with aromatase inhibitors (AIs) as initial endocrine therapy, with concurrent luteinizing hormone-releasing hormone (LHRH) agonist administration required for pre-/perimenopausal patients (Braal et al., 2021). The recommended regimen—600 mg once daily for 21 days followed by a 7-day hiatus (28-day cycle)—is maintained until disease progression or intolerable toxicity (Parati et al., 2022). Pharmacokinetic studies demonstrate rapid absorption (Cmax achieved within 1–4 h post-dose) with 65.8% absolute bioavailability. Ribociclib is extensively metabolized by the liver in humans primarily through CYP3A4. The main metabolic pathways involve oxidation (dealkylation of C and/or N-oxidation, oxidation (-2H)) and different combinations of the various metabolic pathways in them. The I conjugates of phase 1 metabolites of this product include n-acetylation, sulfation, cysteine binding, glycosylation, and glucosylation (Ji et al., 2024).

The MONALEESA-2 trial, a phase III randomized double-blind study, evaluated ribociclib + letrozole versus placebo + letrozole in 668 treatment-naïve postmenopausal women with HR+/HER2- advanced breast cancer. Participants were stratified 1:1, with PFS assessed by investigators using RECIST v1.1 and confirmed via blinded independent central review. At the prespecified interim analysis (80% PFS events), the ribociclib arm demonstrated superior efficacy: median PFS was 25.3 months versus 16.0 months, with 54.7% of ribociclib-treated patients versus 35.9% in the placebo group remaining progression-free at 24 months (Sanò et al., 2022; Büyükkaramikli et al., 2019). The MONALEESA-7 trial, a phase III randomized double-blind study, enrolled 672 pre-/perimenopausal women with HR+/HER2- advanced breast cancer to evaluate ribociclib + endocrine therapy (tamoxifen or non-steroidal aromatase inhibitors [NSAI] + goserelin) versus placebo + endocrine therapy. After 318 PFS events, ribociclib combined with NSAI + goserelin achieved a median PFS of 27.5 months versus 13.8 months for placebo, with the overall cohort (including tamoxifen subgroups) showing 23.8-month vs. 13.0-month median PFS (Tanguy et al., 2018; Shah et al., 2020). The most common adverse reactions to Ribociclib in clinical studies (incidence ≥20%, higher than Placebo group) were neutropenia, infection, nausea, fatigue, diarrhea, leukopenia, vomiting, pain, constipation, hair loss, cough, rash, back pain, anemia, and abnormal liver function tests (Stanciu et al., 2023; Kassem et al., 2018).

### 3.3 Abemaciclib

Abemaciclib, an oral CDK4/6 inhibitor developed by Eli Lilly, received FDA approval in September 2017 and holds distinction as the only CDK4/6 inhibitor approved for both early-stage and advanced HR+/HER2- breast cancer. For early-stage disease, it is indicated in combination with endocrine therapy (tamoxifen or aromatase inhibitors) for adults with lymph node-positive, high-risk recurrence HR+/HER2- breast cancer; and as monotherapy or combined with fulvestrant for HR+/HER2- locally advanced or metastatic disease, including first-line endocrine therapy in postmenopausal women or post-endocrine progression settings (Yang et al., 2022). The standard dose is 150 mg twice daily with continuous dosing, requiring concurrent gonadotropin-releasing hormone agonists in pre-/perimenopausal patients (O'Keefe et al., 2024). Abemaciclib is slowly absorbed, with a peak time of 8 h after the recommended oral dose of 150 mg and an average absolute bioavailability of about 45%. Liver metabolism is the main clearance pathway of Abemaciclib, mainly through cytochrome P450(CYP)3A4 metabolites to hydroxylation, N-demethylation and N-demethylation hydroxyl metabolites, which are active and similar in potency to Abemaciclib (Robert et al., 2019; Martorana et al., 2024).

The MONARCH E trial, a phase III open-label study, evaluated abemaciclib combined with adjuvant endocrine therapy versus endocrine therapy alone in 5,637 high-risk patients with HR+/HER2-, lymph node-positive early breast cancer. With invasive disease-free survival (IDFS) as the primary endpoint, Abemaciclib combined with endocrine therapy significantly improved IDFS, and the 4-year IDFS rate was 85.8% in the combined treatment group and 79.4% in the endocrine therapy group alone, and the 4-year absolute benefit was 6.4% (Martín et al., 2023; Orozco Leal et al., 2023). The MONARCH 3 trial, a phase III randomized double-blind study, evaluated abemaciclib combined with nonsteroidal aromatase inhibitors (NSAI: anastrozole/letrozole) versus NSAI + placebo in 493 postmenopausal women with HR+/HER2- locally advanced or metastatic breast cancer. With investigator-assessed progression-free survival (PFS) by RECIST v1.1 as the primary endpoint, the abemaciclib arm achieved a median PFS of 28.18 months versus 14.76 months, representing a 46% reduction in disease progression or death risk (Tanguy et al., 2018). The MONARCH 2 trial, a phase III randomized double-blind study, evaluated abemaciclib combined with fulvestrant versus fulvestrant + placebo in 669 women with HR+/HER2- locally advanced or metastatic breast cancer. With PFS as the primary endpoint, the abemaciclib arm demonstrated a median PFS of 16.4 months versus 9.3 months, corresponding to a 44.7% reduction in disease progression/death risk and a 7.2-month PFS improvement (El et al., 2021; Sobhani et al., 2019). The most common adverse effects of Abemaciclib in clinical studies include diarrhea, infection, neutropenia, leukopenia, anemia, fatigue, nausea, vomiting, hair loss, and decreased appetite. Among the most common adverse reactions, grade >3 events were less than 5% except for neutropenia, leukopenia and diarrhea (Wekking et al., 2023; Gebbia et al., 2020).

### 3.4 Dalpiciclib

Dalpiciclib, China’s first domestically developed selective CDK4/6 inhibitor by Jiangsu Hengrui Medicine, received NMPA approval in December 2021 for HR+/HER2− recurrent/metastatic breast cancer post-endocrine progression when combined with fulvestrant (Chen and Shen, 2023). In June 2023, its indication expanded to first-line therapy with aromatase inhibitors for HR+/HER2− locally advanced/metastatic disease (Zhao et al., 2023). The recommended regimen is 150 mg once daily under fasting conditions (1 h pre- and post-dose) via a 21-day on/7-day off cycle. Premenopausal patients require concurrent gonadotropin-releasing hormone agonists (Nabieva and Fasching, 2023; Wang et al., 2022). Pharmacokinetically, Dalpiciclib reaches peak plasma concentration at 3.5 h post-dose, with CYP3A4-mediated oxidation serving as the primary metabolic pathway (cyclopentane/piperidine ring modifications), complemented by CYP2C9/2C8-mediated glucuronidation and sulfation (Zhang et al., 2021; Tao et al., 2023).

The phase II SHR6390-III-301 trial evaluated Dalpiciclib, China’s first domestically developed CDK4/6 inhibitor, combined with fulvestrant versus placebo + fulvestrant in 361 patients with HR+/HER2- recurrent/metastatic breast cancer progressing after endocrine therapy. Participants were randomized 2:1, receiving Dalpiciclib + fulvestrant or placebo + fulvestrant until progression or intolerance. Investigator-assessed PFS by RECIST v1.1 demonstrated superiority for Dalpiciclib: median PFS 15.7 vs. 7.2 months (Zhang et al., 2021; Yanke et al., 2019). The most common adverse events (incidence >10%) associated with the use of Dalpiciclib in combination with Fulvestrant in the treatment of recurrent or metastatic breast cancer include decreased neutrophil counts, decreased white blood cell counts, anemia, decreased platelet counts, rash, elevated liver enzymes, nausea, decreased lymphocyte counts, skeletal muscle pain, oral catarrh, fatigue, and elevated serum creatinine. Adverse reactions with severity of grade 3 and above with an incidence of >2% included decreased neutrophil count, decreased white blood cell count, decreased platelet count, decreased lymphocyte count, and anemia (Shi et al., 2024).

### 3.5 Trilaciclib

Trilaciclib, an intravenous CDK4/6 inhibitor developed by G1 Therapeutics, received FDA approval in February 2021 as the first and only myeloprotective agent to mitigate chemotherapy-induced myelosuppression (Crawford et al., 2024). By inducing transient G1 arrest in bone marrow hematopoietic stem/progenitor cells, it shields proliferating hematopoietic lineages from cytotoxic damage during platinum/etoposide regimens for extensive-stage small cell lung cancer (ES-SCLC) in chemotherapy-naïve patients (Goldschmidt et al., 2023). The recommended dose of Trilaciclib is 240 mg/m^2^, which should be completed by intravenous infusion for 30 min within 4 h before chemotherapy administration on the same day. When Trilaciclib is given for several consecutive days, the interval between administration should not exceed 28 h (Roskoski, 2024). The average terminal half-life of Trilaciclib is about 14 h, and clearance is estimated at 158L/hr. Trilaciclib is metabolized extensively and is excreted mainly through the fecal pathway (Dhillon, 2021).

The G1T28-02 trial, a randomized double-blind study, evaluated trilaciclib’s myeloprotective efficacy in 75 chemotherapy-naïve extensive-stage small cell lung cancer (ES-SCLC) patients receiving first-line etoposide/carboplatin (E/P). Participants were randomized to receive trilaciclib or placebo, with carboplatin (AUC 5) administered on day 1 and etoposide on days 1–3. Trilaciclib significantly reduced hematologic toxicity: cycle one severe neutropenia duration decreased from 3 days (placebo) to 0 days (trilaciclib), with incidence rates of 5.1% vs. 42.1%. Hemoglobin reduction ≥grade 3 occurred in 10% vs. 18% of patients, while erythrocyte transfusion requirements decreased from 1.9 to 0.5 events/100 weeks. Erythropoiesis-stimulating agent use was halved (3% vs. 5%) (Crawford and Oswalt, 2023; Falandry et al., 2023). Adverse effects of Trilaciclib in clinical studies include injection site reactions, including phlebitis and thrombophlebitis, acute drug hypersensitivity, and interstitial lung disease/pulmonary inflammation. Other most common adverse events include fatigue, hypocalcemia, hypokalemia, hypophosphatemia, elevated aspartate aminotransferase, headache, and infectious pneumonia (Dubey et al., 2024; Roberts et al., 2020).

## 4 Resistance of CDK4/6 inhibitors

**FIGURE 2:**
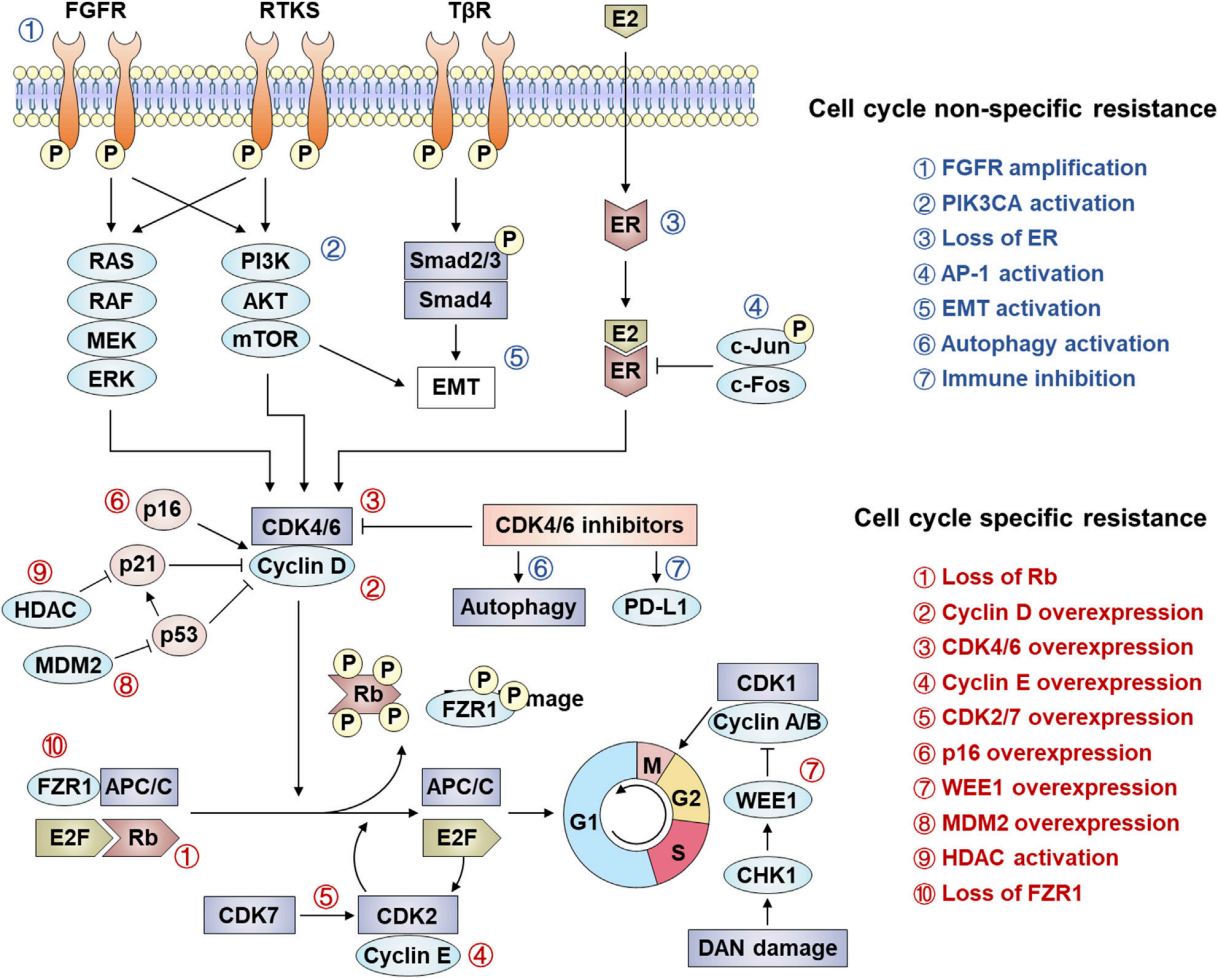
Mechanism of resistance to CDK4/6 inhibition. Several mechanisms of resistance to CDK 4/6 signaling have been established, including cell cycle specific resistance and cell cycle non-specific resistance.

CDK4/6 inhibitors significantly suppress disease progression in HR+/HER2- breast cancer, demonstrating marked therapeutic synergy when combined with endocrine therapies. However, not all patients have a good clinical benefit from this class of drugs, and most patients will develop acquired resistance after receiving CDK4/6 inhibitor treatment (Zhang et al., 2025). Elucidating resistance mechanisms—particularly those enabling tumor adaptation through cell cycle rewiring or bypass signaling—has consequently emerged as a critical research priority. Current models classify resistance into two broad categories: cell cycle specific resistance (changes in cell cycle progression) and cell cycle non-specific resistance (changes in upstream signaling pathways) (Figure 2) (Foffano et al., 2025).

### 4.1 Cell cycle specific resistance

#### 4.1.1 Absence of Rb and overexpression of cyclin D, CDK4 and CDK6

CDK4/6 inhibitors exert their therapeutic effects by targeting the CDK4/6-cyclin D-Rb axis, making alterations in core components of this axis—including Rb loss, CDK4/6 overexpression, or cyclin D upregulation—primary drivers of resistance. Rb, a critical mediator of CDK4/6 inhibitor efficacy, determines therapeutic sensitivity, as these agents potently suppress Rb-positive tumors (e.g., breast, colorectal, and lung cancers) but show minimal activity in Rb-deficient malignancies (Iacovacci et al., 2025). Clinically, acquired *RB1* mutations or deletions emerge as predominant resistance mechanisms, evidenced by polyclonal Rb alterations in metastatic breast cancer patients progressing on palbociclib and preclinical models demonstrating Rb loss-triggered bypass signaling via E2F hyperactivation or CDK2-cyclin E axis compensation (Malumbres et al., 2004; Condorelli et al., 2018; O'Leary et al., 2018). Concurrently, CDK4/6 overexpression reduces drug efficacy, as shown in CDK4-amplified alveolar rhabdomyosarcoma and glioma models, while CDK6 upregulation in resistant MCF-7 cells restores Rb phosphorylation capacity (Olanich et al., 2015; Cen et al., 2012; Ogata et al., 2021; Yang et al., 2017). Further, Cyclin D dysregulation further complicates resistance, with *CCND1* amplification occurring in ∼50% of breast cancers and enabling persistent CDK4/6-Rb signaling despite inhibitor exposure (Finn et al., 2009). Understanding these resistance mechanisms directly informs therapeutic strategies: Rb status assessment could guide patient stratification, while CDK4/6 or cyclin D overexpression may predict benefit from dose escalation or combination therapies (e.g., CDK2 inhibitors). Monitoring acquired *RB1* mutations through liquid biopsies enables real-time adaptation of treatment regimens, emphasizing the need for dynamic biomarker-driven approaches to overcome resistance and prolong clinical responses.

#### 4.1.2 Overexpression of cyclin E, CDK2, CDK7 and p16

Resistance to CDK4/6 inhibitors frequently arises from compensatory upregulation of alternative cell cycle drivers, including Cyclin E-CDK2 complexes, CDK7, and dysregulated p16. While the canonical CDK4/6-cyclin D-Rb axis governs G1/S progression, Cyclin E1/E2 (encoded by *CCNE1/2*) overexpression enables CDK2-mediated Rb phosphorylation, bypassing CDK4/6 inhibition to sustain proliferation (Doostan et al., 2017; Turner et al., 2019). Clinical relevance is underscored by the PALOMA-3 trial, where low *CCNE1* expression correlated with enhanced palbociclib efficacy, suggesting Cyclin E-CDK2 activity as a resistance biomarker (Cristofanilli et al., 2016). Proteolytic cleavage of Cyclin E generates low molecular weight isoforms (LMW-E) that hyperphosphorylate Rb, independently predicting breast cancer recurrence risk (Caruso et al., 2018). Concurrently, CDK7—a master regulator of CDK1/2/4/6 activation—promotes resistance through kinase cascade amplification, while p16 INK4A deficiency reduces CDK4 inhibition efficacy by disrupting endogenous cell cycle control (Schachter et al., 2013; Bhurta and Bharate, 2022; Liu et al., 2022). These resistance mechanisms highlight actionable therapeutic opportunities: *CCNE1* expression profiling could stratify patients for CDK2 inhibitor combinations, while CDK7 targeting (e.g., THZ1) may overcome compensatory kinase activation. Detection of LMW-E or p16 loss via tumor sequencing enables dynamic treatment adaptation, emphasizing the need for real-time molecular monitoring to guide sequential therapies and prevent resistance-driven relapse.

#### 4.1.3 Overexpression of WEE1 and MDM2

Resistance to CDK4/6 inhibitors is further mediated by dysregulation of cell cycle checkpoints and p53 signaling, exemplified by WEE1 and MDM2 overexpression. WEE1, a serine/threonine kinase critical for G2/M checkpoint control via DNA replication fidelity, is upregulated in breast cancer, leukemia, and melanoma. Its overexpression confers CDK4/6 inhibitor resistance by enabling G2/M progression despite CDK4/6 blockade, though WEE1 inhibition with adavosertib (AZD1775) restores sensitivity and synergizes with CDK4/6 inhibitors (Matheson et al., 2016; Francis et al., 2017). Concurrently, MDM2—overexpressed in 20%–30% of breast cancers—antagonizes p53-mediated cell cycle arrest by degrading p53 and suppressing its activation of p21 CIP1, thereby bypassing CDK4/6 inhibitor-induced G1 arrest (Efeyan et al., 2007; Portman et al., 2021). Preclinical studies demonstrate that combining MDM2 inhibitors (e.g., CGM097) with CDK4/6 inhibitors and fulvestrant overcomes dual resistance to endocrine and CDK4/6-targeted therapies (Laroche-Clary et al., 2017). Targeting WEE1 or MDM2 provides a rational strategy to reverse CDK4/6 inhibitor resistance: WEE1 expression profiling could identify candidates for adavosertib combinations, while MDM2 inhibition restores p53-dependent cell cycle control in p53-wild-type tumors. Liquid biopsy monitoring for WEE1/MDM2 amplifications during treatment enables adaptive therapy switching, emphasizing biomarker-guided combinatorial approaches to prolong therapeutic efficacy and prevent relapse.

#### 4.1.4 Activation of HDACs and loss of FZR1

Epigenetic dysregulation, particularly through histone deacetylase (HDAC) activation and FZR1 loss, constitutes a key mechanism of CDK4/6 inhibitor resistance. HDACs drive resistance by deacetylating histones (e.g., H3K27ac reduction) to silence tumor suppressor genes and disrupt p21-mediated cell cycle arrest, while HDAC5 deletion in breast cancer upregulates oncogenic transcription via hyperacetylated chromatin states (Rosato and SJJoCO, 2003; Zhou et al., 2021). Notably, HDACs recruit Rb through LXCXE motifs to reinforce epigenetic repression, creating a feedforward loop that sustains proliferation despite CDK4/6 inhibition. Concurrently, FZR1 deficiency disrupts anaphase-promoting complex/cyclosome (APC/C) function, preventing SKP2 degradation and stabilizing CDK2/4/6 kinases. Phosphorylated FZR1 inactivation by cyclin D-CDK4/6 further impairs APC/C-mediated cell cycle control, enabling tumors to bypass G1/S checkpoint dependence—a resistance mechanism amplified by combined FZR1/Rb loss (Ramanujan and Tiwari, 2016; Turner et al., 2010). Targeting HDACs with inhibitors (e.g., panobinostat) alongside CDK4/6 blockade may reverse epigenetic-driven resistance, particularly in HDAC5-deficient tumors. FZR1 expression profiling could identify candidates for APC/C activators or CDK2 inhibitors, while liquid biopsies monitoring H3K27ac levels or FZR1 mutations enable dynamic therapy adaptation. These strategies exemplify precision oncology’s potential to convert resistance mechanisms into actionable therapeutic vulnerabilities, extending durable responses in CDK4/6 inhibitor-treated patients.

### 4.2 Cell cycle non-specific resistance

#### 4.2.1 Activation of FGFR

Fibroblast growth factor receptor (FGFR) is a family of receptor tyrosine kinases expressed on cell membranes, whose signaling pathway is involved in key biological processes such as cell proliferation and differentiation, and maintenance of cell survival (Dai et al., 2019; Turner and Grose, 2010). Aberrant activation of FGFR signaling—a receptor tyrosine kinase family encompassing FGFR1-4—drives therapeutic resistance in hormone receptor-positive breast cancer by bypassing CDK4/6 inhibitor-induced cell cycle arrest. FGFR alterations (mutations/amplifications) occur in 7.1% of solid tumors, with clinical studies (PALOMA-3, MONALEESA-2) demonstrating FGFR amplification correlates with shortened progression-free survival (PFS) and dual resistance to CDK4/6 inhibitors/endocrine therapy via ctDNA analysis (Helsten et al., 2016; Formisano et al., 2019; O'Leary et al., 2021). Mechanistically, FGFR activation converges with FGF2 to hyperactivate PI3K/AKT and RAS/RAF/MEK/ERK pathways, sustaining proliferation while downregulating progesterone receptors to diminish endocrine sensitivity (Du et al., 2023). Notably, FGFR signaling induces epigenetic suppression of cell cycle inhibitors, enabling tumor cell survival despite CDK4/6 blockade. Current clinical trials are evaluating FGFR inhibitors (e.g., erdafitinib) to reverse this resistance phenotype (Turner et al., 2010). FGFR amplification status should be incorporated into resistance biomarker panels to guide combination therapies (e.g., CDK4/6 inhibitors + FGFR/PI3K inhibitors). Longitudinal ctDNA monitoring for emergent FGFR alterations during treatment enables timely therapeutic adaptation, while progesterone receptor expression tracking may predict FGFR-mediated endocrine resistance. These strategies exemplify the critical need for pathway-informed sequential therapy to counteract adaptive tumor evolution and prolong clinical benefit.

#### 4.2.2 Activation of the PI3K/AKT/mTOR signalling pathway

The PI3K/AKT/mTOR signaling pathway, critically involved in tumor growth, proliferation, metabolism, and migration, represents one of the most frequently dysregulated pathways in breast cancer therapeutics (Liu et al., 2024). In CDK4/6 inhibitor-resistant breast cancer cells, tumor growth shifts dependency from hormone receptor (HR) signaling to hyperactivated PI3K/AKT/mTOR cascades. This is exemplified by *PIK3CA* mutations in 30%–40% of HR+ breast cancers, driving constitutive PI3K pathway activation and conferring dual resistance to CDK4/6 inhibitors and endocrine therapies (Abu-Khalaf et al., 2023). Notably, mTOR-mediated phosphorylation of Rb and E2F activation in resistant cells establishes CDK4/6-independent cell cycle progression, which can be reversed through dual mTORC1/2 inhibition (Browne et al., 2024). Furthermore, PI3K/mTOR inhibitors counteract cyclin D1/CDK4 overexpression—a hallmark of acquired resistance—by upregulating the translation repressor 4E-BP1, as demonstrated in preclinical models by Cai et al. (Cai et al., 2023). These findings position 4E-BP1 as a potential predictive biomarker and support rational combination therapies pairing CDK4/6 inhibitors with PI3K/mTOR pathway antagonists to circumvent resistance (O'Brien et al., 2020). Routine *PIK3CA* mutation screening should be integrated into resistance management protocols to identify candidates for PI3K/mTOR inhibitor combinations. Monitoring 4E-BP1 expression levels could further stratify patients likely to benefit from translational repression strategies. These approaches exemplify the paradigm of targeting resistance pathways through biomarker-guided therapy sequencing, ultimately extending the clinical utility of CDK4/6 inhibitors in advanced breast cancer.

#### 4.2.3 Loss of HR expression and overexpression of AP-1

Resistance to CDK4/6 inhibitors in breast cancer is increasingly linked to hormonal pathway rewiring and transcriptional reprogramming, exemplified by hormone receptor (HR) loss and AP-1 overexpression. Preclinical models and clinical cohorts reveal that ∼33% of patients exhibit ER/PR expression loss after CDK4/6 inhibitor progression, with *ESR1* mutations—observed in 14.7%–19.2% of PALOMA-3 trial participants—driving ligand-independent ER activation to bypass therapeutic suppression (Mo et al., 2022; Jeselsohn et al., 2015; Hortobagyi et al., 2018; Bardia and Hurvitz, 2018). Concurrently, AP-1 (c-Fos/c-Jun heterodimer) overexpression upregulates cyclin D1 transcription while suppressing ER signaling, enabling dual resistance to CDK4/6 inhibitors and endocrine therapies. In MCF-7 models, c-Jun-mediated cyclin D1 amplification and ER antagonism correlate with palbociclib/tamoxifen resistance, whereas AP-1 blockade synergizes with CDK4/6 inhibition to restore therapeutic efficacy (Ye et al., 2014; Fu et al., 2022; Pandey et al., 2019). Routine *ESR1* mutation screening and AP-1 activity profiling could stratify patients for targeted interventions: *ESR1*-mutant tumors may benefit from selective ER degraders (SERDs), while AP-1-driven resistance warrants exploration of BET or MEK inhibitors in combination with CDK4/6 blockade. Longitudinal monitoring of HR expression and AP-1 biomarkers via liquid biopsies enables adaptive therapy switching, transforming resistance mechanisms into actionable targets to prolong disease control in advanced breast cancer.

#### 4.2.4 Abnormal expression of EMT/TGF-β/Smad3

Epithelial-mesenchymal transition (EMT) and TGF-β/Smad3 signaling axis dysregulation constitute critical drivers of CDK4/6 inhibitor resistance, enabling tumor cell plasticity and therapeutic evasion. CDK4/6 inhibition paradoxically activates TGF-β signaling, which phosphorylates Smad2/3 to form transcriptional complexes with Smad4, inducing EMT transcription factors (e.g., Snail, Twist) and PI3K/AKT/mTOR-mediated mesenchymal reprogramming (Singh and Settleman, 2010; Du and Shim, 2016; Lamouille et al., 2014). Concurrently, Smad3—a tumor suppressor that enforces G1 arrest—is functionally inactivated in resistance models through CDK2-cyclin E-mediated phosphorylation, bypassing Rb-E2F suppression to restore cell cycle progression (Heldin and AJJoCS, 2005). Preclinical evidence demonstrates that Smad3 reconstitution or CDK2 inhibition reverses trastuzumab resistance by blocking Smad3 hyperphosphorylation, while CDK4/6 inhibitor resistance correlates with CDK2-cyclin E axis activation and Smad3 suppression (Zelivianski et al., 2010; Yang et al., 2008). These findings suggest that EMT/TGF-β/Smad3 crosstalk establishes a dual resistance mechanism: TGF-β-driven mesenchymal adaptation and CDK2-mediated Smad3 inactivation. Targeting EMT/TGF-β (e.g., galunisertib) or restoring Smad3 activity could re-sensitize resistant tumors to CDK4/6 inhibitors. Liquid biopsy profiling for EMT markers (e.g., circulating vimentin) or TGF-β pathway components may identify candidates for combination therapies, while Smad3 phosphorylation status could guide CDK2 inhibitor coadministration. These strategies highlight the need to disrupt compensatory signaling plasticity, transforming resistance vulnerabilities into precision therapeutic opportunities for metastatic breast cancer.

#### 4.2.5 Abnormal autophagy activation and immune-related pathways

CDK4/6 inhibitor resistance is intricately linked to aberrant autophagy activation and immune pathway dysregulation, reflecting the dual role of autophagy in tumor biology. While autophagy initially suppresses tumorigenesis by maintaining cellular homeostasis, it evolves into a pro-survival mechanism in advanced cancers, enabling resistance by reversing CDK4/6 inhibitor-induced G1 arrest through reactive oxygen species (ROS) scavenging and metabolic adaptation (Vijayaraghavan et al., 2017; Gong et al., 2024). Transcriptomic profiling of palbociclib-resistant MCF-7 cells reveals upregulated autophagy-related genes and increased autophagosome formation, underscoring ER+ breast cancer’s reliance on autophagy to evade CDK4/6 blockade (Lanceta et al., 2020; Soria-Bretones et al., 2022). Paradoxically, although CDK4/6 inhibitors enhance antitumor immunity via T-cell activation, resistant tumors exhibit mutations in immune-regulatory genes (e.g., *NCOR1*, *MUC4*, *MUC16*), leading to immune pathway inactivation and diminished T-cell cytotoxicity (Pandey et al., 2021). Concurrent PD-L1 upregulation by CDK4/6 inhibitors further complicates this interplay, suggesting that immune checkpoint blockade may counteract resistance by leveraging heightened tumor immunogenicity (Zhang et al., 2018). Targeting autophagy (e.g., chloroquine) or combining CDK4/6 inhibitors with PD-1/PD-L1 inhibitors offers a rational strategy to overcome resistance. Liquid biopsy monitoring of autophagy markers (LC3B) and immune gene mutations could enable early detection of resistance, while PD-L1 expression dynamics may guide immunotherapy sequencing. These approaches highlight the imperative to co-target pro-survival and immune-evasion pathways, transforming resistance mechanisms into therapeutic vulnerabilities for precision oncology.

## 5 Combination therapy based on CDK4/6 inhibitors

**FIGURE 3:**
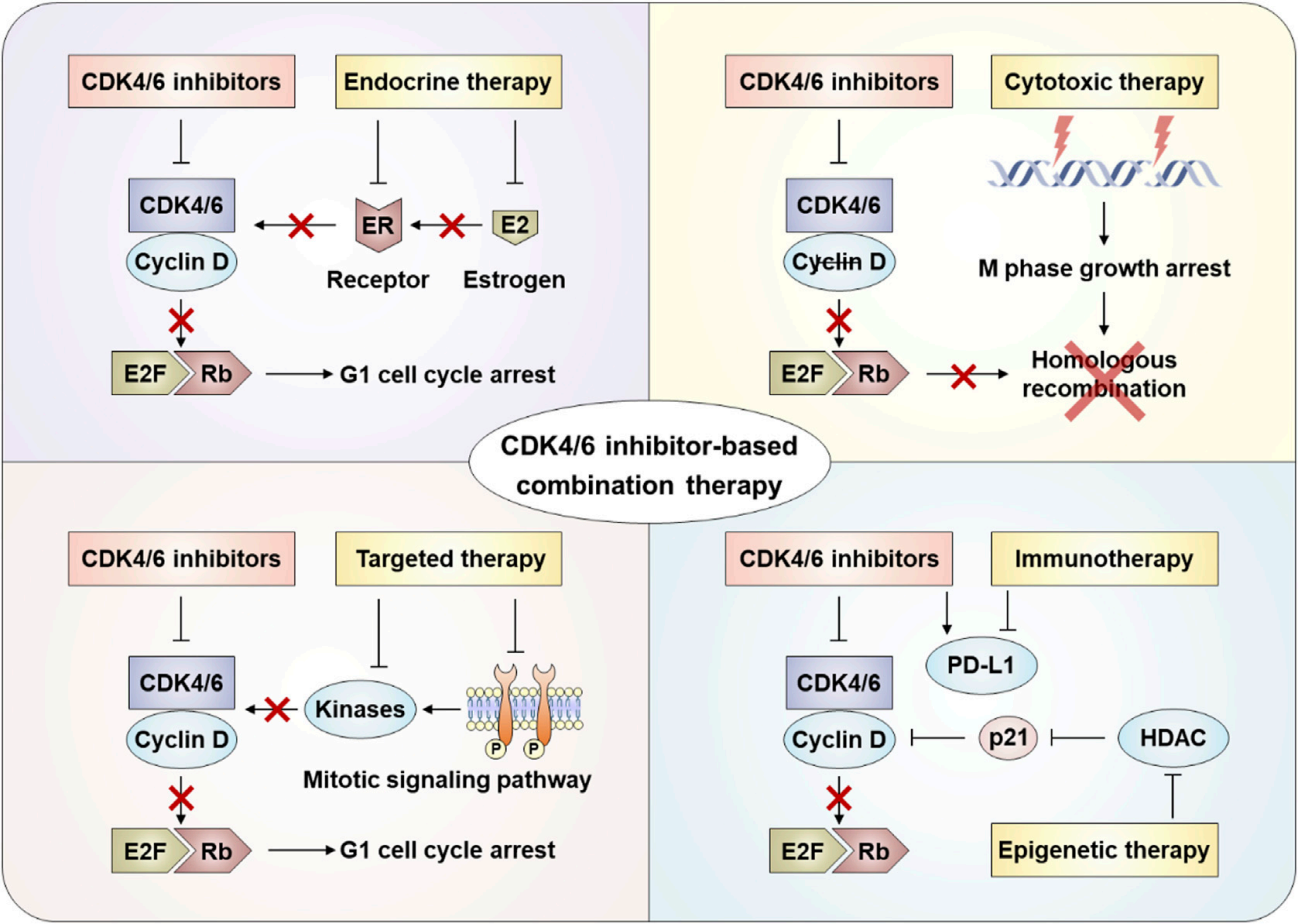
Combination strategies after CDK4/6 inhibitor resistance.

After the progresses of CDK4/6 inhibitors treatment, different coping strategies are required for different resistance mechanisms. The reasons for drug resistance in patients are multifaceted, and the molecular mechanism is very complex. Treatment options after CDK4/6 inhibitor resistance remain limited and controversial, and there is no optimal clinical management path. At present, the main treatment options after CDK4/6 inhibitor resistance include replacing other endocrine drugs with CDK4/6 inhibitors, selecting cytotoxic drugs, and combined targeted therapy, immunotherapy, or epigenetic therapy (Figure 3) (Sahin et al., 2025; Abdullaeva et al., 2025).

### 5.1 Combined with endocrine therapy

CDK4/6 inhibitors combined with endocrine therapy (aromatase inhibitors or fulvestrant) represent the standard-of-care for HR+HER2- advanced breast cancer, demonstrating significant improvements in progression-free survival (PFS) and overall survival (OS) across first- and later-line settings (Wang et al., 2020; Howie et al., 2019). This therapeutic synergy stems from multi-level crosstalk between ER signaling and the CDK4/6-cyclin D-Rb axis: (1) CCND1 (cyclin D1) is transcriptionally regulated by ER (Valerio et al., 2025); (2) ER activates E2F-driven proliferation independently of estrogen (Miller et al., 2011); and (3) cyclin D1 enhances ER transcriptional activity in a CDK-independent manner (Zwijsen et al., 1997). While this dual blockade effectively suppresses tumor growth, acquired resistance inevitably arises, prompting exploration of post-progression strategies such as switching CDK4/6 inhibitors (e.g., palbociclib → ribociclib) or altering endocrine partners (e.g., fulvestrant → exemestane) (Schoninger and Blain, 2020; Harbeck et al., 2025). The phase II MAINTAIN trial (NCT02632045) demonstrated modest benefit with ribociclib rechallenge (median PFS: 5.3 vs. 2.76 months; placebo), whereas the PACE study (NCT03147287) showed no PFS improvement with palbociclib continuation (4.8 vs. 4.6 months) post-progression, underscoring heterogeneous responses influenced by Rb loss, cyclin E1 overexpression, and ESR1 mutations [147–149].

The concept of CDK4/6 inhibitor rechallenge is exemplified by the phase II MAINTAIN trial (NCT02632045), which randomized 120 patients progressing on prior CDK4/6 inhibitors (84% palbociclib-treated) to fulvestrant (83%) or exemestane (17%) ± ribociclib. The ribociclib cohort achieved superior median PFS (5.3 vs. 2.76 months), with 25% vs. 7% of patients remaining progression-free at 12-month follow-up (Kalinsky et al., 2022). In contrast, the PACE trial (NCT03147287) evaluated palbociclib continuation post-resistance (91% prior palbociclib exposure), randomizing 120 patients to fulvestrant alone, fulvestrant + palbociclib, or fulvestrant + palbociclib + avelumab. No PFS (4.8 vs. 4.6 months) or OS (27.5 vs. 24.46 months) benefit was observed with palbociclib rechallenge (Mayer et al., 2018). Biomarker-driven insights emerged from the BioPER study, where palbociclib rechallenge in HR+/HER2- patients yielded median PFS of 1.9 months versus 6.7 months in subgroups with Rb loss, cyclin E1 amplification, or ESR1 mutations (Albanell et al., 2023). These findings informed the CSCO 2022 Breast Cancer Guidelines to cautiously recommend CDK4/6 inhibitor switching post-progression, albeit with low evidence strength due to limited trial sizes. However, implementing rechallenge strategies faces critical challenges. First, overlapping toxicity profiles (e.g., neutropenia from sequential CDK4/6 inhibitors) may limit tolerability. Second, clonal evolution under therapy pressure generates heterogeneous resistance mechanisms (e.g., Rb loss vs. cyclin E1 amplification), necessitating dynamic biomarker monitoring. Finally, the lack of standardized criteria for patient selection—particularly in distinguishing true CDK4/6 inhibitor resistance from endocrine therapy failure—compromises therapeutic precision. Addressing these limitations requires adaptive trial designs integrating real-time genomic profiling and combinatorial approaches targeting non-overlapping resistance pathways.

### 5.2 Combined with cytotoxic therapy

Cytotoxic therapies (e.g., radiotherapy, chemotherapy) exert antitumor effects by disrupting critical cellular processes such as DNA replication and protein synthesis in cancer cells (Kornepati et al., 2023). Combining chemotherapy with CDK4/6 inhibitors post-resistance shows therapeutic potential due to their non-overlapping resistance mechanisms. Mechanistically, CDK4/6 inhibitors induce tumor cell cycle arrest and apoptosis, while chemotherapy-induced DNA damage may sensitize tumors to cell cycle-targeted therapies (Bisi et al., 2016; He et al., 2017). However, chemotherapy’s dose-limiting hematologic toxicities (e.g., bone marrow suppression) often restrict its clinical utility. Notably, co-administration of CDK4/6 inhibitors may protect hematopoietic stem cells from chemotherapy-induced myelotoxicity (Bisi et al., 2016; He et al., 2017). Preclinical models demonstrate enhanced efficacy when microtubule stabilizers or DNA-damaging agents precede CDK4/6 inhibition, as suppressed E2F target gene expression impairs homologous recombination-mediated DNA repair (Dean et al., 2012; Salvador-Barbero et al., 2020). Additionally, Rb-independent effects of CDK4/6 inhibitors—such as blocking phosphorylation of FOXM1 and FOXO3—further compromise DNA repair capacity post-chemotherapy or radiotherapy across diverse tumor types (Hashizume et al., 2016).

For patients developing resistance to first-line CDK4/6 inhibitors, switching endocrine therapies or reintroducing CDK4/6 inhibitors may prove ineffective in those with Rb loss, CCNE1 amplification, or ESR1 mutations (Turner et al., 2018; Xi et al., 2019). Here, cytotoxic therapies could eliminate clones harboring these resistance drivers, potentially resensitizing tumors to CDK4/6 inhibitors. Thus, sequential administration of cytotoxic therapy followed by CDK4/6 inhibitor rechallenge represents a promising strategy. Clinical practice reflects this rationale, with chemotherapy being the most common post-CDK4/6 inhibitor progression therapy (Park et al., 2019). In the PALOMA-3 trial, capecitabine, eribulin, and nab-paclitaxel dominated chemotherapy regimens for palbociclib-fulvestrant-resistant patients. Intriguingly, patients with poor prior palbociclib response achieved longer median progression-free survival (mPFS) on subsequent chemotherapy than those with initial sensitivity (Li et al., 2021). Similarly, a U.S. real-world study reported 35.6% of post-CDK4/6 inhibitor patients receiving second-line chemotherapy, primarily capecitabine and taxanes (Princic et al., 2019). These findings suggest that chemotherapy may circumvent endocrine therapy resistance pathways linked to CDK4/6 inhibitor failure. However, this sequential approach faces significant challenges. First, cumulative hematologic toxicity from chemotherapy and CDK4/6 inhibitors (both myelosuppressive) may limit therapeutic feasibility. Second, tumor heterogeneity enables rapid evolution of alternative resistance mechanisms, undermining sustained efficacy. Finally, the lack of validated biomarkers to identify patients likely to benefit from cytotoxic-CDK4/6 inhibitor sequencing hinders personalized treatment optimization. Addressing these limitations requires prospective studies correlating genomic instability patterns with therapeutic responses.

### 5.3 Combined with targeted therapy

Extensive preclinical evidence demonstrates synergistic effects between CDK4/6 inhibitors and mitotic signaling pathway inhibitors across multiple tumor models. CDK4/6 inhibition exhibits bidirectional crosstalk with oncogenic kinases, including components of the PI3K-AKT and MAPK pathways, as well as upstream receptors such as EGFR, HER2, and fibroblast growth factor receptors (Goel et al., 2016; Hallin et al., 2020). Mechanistically, sustained CDK4/6 activity can mediate resistance to kinase-targeted therapies, while hyperactivation of mitotic signaling pathways conversely drives resistance to CDK4/6 inhibitors—phenomena frequently observed concurrently (Lou et al., 2019; Kwong et al., 2012). A hallmark example is compensatory CDK2 activation through enhanced Rb phosphorylation via upstream signaling, enabling S-phase entry despite CDK4/6 blockade. This reciprocal resistance underscores the therapeutic rationale for combining CDK4/6 inhibitors with upstream kinase inhibitors tailored to tumor-specific signaling aberrations (Formisano et al., 2019; Heilmann et al., 2014).

PI3K/AKT/mTOR pathway activation constitutes a key endocrine therapy resistance mechanism, prompting clinical investigations into combining pathway inhibitors with endocrine therapy post-CDK4/6 inhibitor progression (Vora et al., 2014). The SOLAR-1 trial demonstrated that in PIK3CA-mutated, CDK4/6 inhibitor-resistant HR+/HER2− metastatic breast cancer, fulvestrant combined with the PI3K inhibitor alpelisib achieved a median progression-free survival (mPFS) of 5.5 months versus 1.8 months with fulvestrant alone (André et al., 2021). Similarly, the CAPItello-291 study reported doubled mPFS (7.2 vs. 3.6 months) for AKT inhibitor capivasertib plus fulvestrant in advanced HR+/HER2− breast cancer, with consistent benefits observed in CDK4/6 inhibitor-pretreated subgroups (5.5 vs. 2.6 months) (Turner et al., 2023). The TRINITI-1 trial further supported this strategy, showing a 41.1% clinical benefit rate in CDK4/6 inhibitor-resistant patients receiving ribociclib, everolimus (mTOR inhibitor), and exemestane (Bardia et al., 2021). These findings position PI3K/AKT/mTOR inhibitors as viable options for patients progressing on frontline CDK4/6 inhibitor-endocrine therapy combinations. Emerging strategies also target kinase-driven resistance mechanisms, as exemplified by the Phase Ib trial (NCT03238196) evaluating palbociclib/fulvestrant with erdafitinib (pan-FGFR inhibitor) in FGFR-amplified, CDK4/6 inhibitor-resistant cases, which demonstrated manageable safety and preliminary efficacy. However, implementing such combination therapies faces substantial challenges. First, overlapping metabolic toxicities (e.g., hyperglycemia from PI3K/AKT inhibitors and gastrointestinal/hepatic effects from CDK4/6 inhibitors) complicate dose optimization and tolerability. Second, dynamic tumor evolution may bypass dual pathway inhibition through compensatory mechanisms, such as MAPK pathway reactivation. Finally, the absence of predictive biomarkers beyond PIK3CA mutations limits patient stratification, risking unnecessary toxicity in non-responders. Addressing these limitations requires longitudinal biomarker monitoring and adaptive trial designs to match therapies with evolving tumor dependencies.

### 5.4 Combined with immunotherapy

The immune escape caused by increased programmed death receptor-ligand 1 (PD-L1) expression following CDK4/6 inhibitor treatment is a recognized contributor to treatment resistance. Two primary mechanisms underlie this drug resistance phenomenon. First, CDK4/6 inhibitors suppress CDK4-Cyclin D-mediated SPOP phosphorylation, thereby preventing ubiquitination-dependent degradation of PD-L1 protein and leading to its intracellular accumulation (Dougan et al., 2019). Second, studies indicate that CDK4/6 inhibition upregulates the oncoprotein Myc, which directly binds to the PD-L1 gene promoter to enhance its transcriptional activation (Tarrado-Castellarnau et al., 2017; Casey et al., 2018; Casey et al., 2016). Both pathways ultimately promote tumor immune evasion through PD-L1 overexpression. Preclinical studies demonstrate synergistic antitumor effects of combined CDK4/6 and PD-L1/PD-1 inhibitors, including enhanced antigen presentation, T-cell activation, and cell cycle arrest (Zhang et al., 2018). Consequently, evaluating the clinical efficacy of immune checkpoint inhibitors following CDK4/6 inhibitor resistance represents a critical area of current clinical research.

Monotherapy with immune checkpoint inhibitors remains uncommon in HR+/HER2- metastatic breast cancer, but combination therapy involving CDK4/6 inhibitors and immunotherapy is being actively investigated for patients progressing on CDK4/6 inhibitors. Preclinical models revealed that abemaciclib combined with PD-1 blockade significantly improved antitumor immune responses *in vivo* (Zhang et al., 2018). The Phase Ib JPCE trial further established the manageable safety profile of this approach in pretreated HR+/HER2- metastatic breast cancer patients (Masuda et al., 2020). Given the emerging resistance mechanisms to CDK4/6 inhibitors, novel immunotherapy combinations are both scientifically justified and clinically imperative, with multiple related trials currently underway. However, several challenges and limitations must be addressed in implementing combination therapies. First, overlapping toxicities (e.g., hematologic adverse events from CDK4/6 inhibitors and immune-related adverse events from checkpoint inhibitors) may limit treatment tolerability. Second, patient selection remains problematic due to the lack of validated biomarkers predicting synergistic efficacy. Additionally, tumor heterogeneity and dynamic evolution of resistance mechanisms during therapy could undermine long-term responses. These biological and clinical complexities highlight the need for robust translational research alongside clinical trials to optimize therapeutic strategies.

### 5.5 Combined with epigenetic therapy

Epigenetic modifications regulate gene expression levels, with histone deacetylase (HDAC)—a key enzyme in histone modification processes—being frequently overexpressed in various cancers (Zhou et al., 2021). This overexpression positions HDAC inhibitors as promising agents in oncology. These inhibitors exert multifaceted antitumor effects, including cell cycle arrest, induction of differentiation/apoptosis/autophagy, senescence, and immunomodulation, by altering histone acetylation dynamics (Falkenberg and Johnstone, 2014; Liao et al., 2018). Beyond conventional therapeutic combinations, emerging insights into CDK4/6 inhibitor-induced epigenetic reprogramming of cancer cells have spurred interest in leveraging this mechanism for synergistic therapeutic strategies. Preclinical studies have indeed demonstrated enhanced antitumor activity when combining CDK4/6 inhibitors with HDAC-targeting agents (Ge et al., 2020).

Chidamide, a selective benzamide-class HDAC inhibitor, is clinically approved for breast cancer treatment. In the Phase III ACE trial involving 365 HR+/HER2- postmenopausal metastatic breast cancer patients with progression after ≥1 endocrine therapy (adjuvant, neoadjuvant, or metastatic), Chidamide combined with exemestane achieved a median progression-free survival (PFS) of 7.4 months, significantly surpassing the 3.8-month PFS in the placebo-exemestane cohort (Jiang et al., 2019). Real-world analyses of CDK4/6 inhibitor-resistant populations further illuminate its potential: among 200 HR+/HER2− metastatic breast cancer patients, a subgroup of 21 receiving Chidamide-based regimens post-progression attained a median PFS of 2.6 months (Li et al., 2021). Another observational study of 44 similar patients reported an overall median PFS of 2.0 months, though those initiating Chidamide immediately after CDK4/6 inhibitor failure reached 4.5 months (Zhou et al., 2022). These data suggest that early Chidamide intervention post-CDK4/6 inhibitor progression may optimize efficacy, though variables like prior treatment lines, metastatic burden, and therapeutic history complicate outcome interpretation, necessitating prospective validation. Integrating HDAC inhibitors with CDK4/6-targeted therapies faces significant challenges. First, overlapping toxicities—such as hematologic adverse events from CDK4/6 inhibitors and gastrointestinal/hematologic effects of HDAC inhibitors—may compromise treatment adherence. Second, epigenetic plasticity and tumor heterogeneity could drive adaptive resistance to combination regimens. Finally, the lack of predictive biomarkers to identify patients most likely to benefit from such combinations hinders personalized therapeutic optimization. Addressing these limitations requires mechanistic studies correlating epigenetic modulation with clinical responses and innovative trial designs to evaluate sequential versus concurrent administration strategies.

## 6 Summary and prospect

CDK4/6 inhibitors combat malignant tumors by regulating cell cycle, changing tumor microenvironment, triggering antitumor immunity and other mechanisms. CDK4/6 inhibitors dramatically change the treatment landscape for patients with HR-positive, HER2-negative advanced breast cancer. All drugs approved by the FDA and NMPA (Palbociclib, Ribociclib, Abemaciclib, and Dalpiciclib) have improved outcomes and acceptable toxicity compared to endocrine therapy alone. Unfortunately, drug resistance is an unavoidable problem. The molecular mechanism of CDK4/6 inhibitor resistance is very complex, and it is important to identify the mechanism of resistance for the next treatment. On the one hand, abnormalities in key molecules of the CDK4/-Cyclin D-Rb regulatory axis can lead to drug resistance. On the other hand, abnormalities in upstream regulators of cell cycle pathways are also common causes of drug resistance. At present, treatment options after CDK4/6 inhibitor resistance remain limited and controversial, and there is no clear and systematic treatment strategy internationally. The treatment options after CDK4/6 inhibitor resistance can be basically divided into three categories: one is to switch to other endocrine drugs combined with CDK4/6 inhibitors, the second is to use combined targeted therapy, and the third is to switch to cytotoxic drugs for chemotherapy. Further, with the in-depth study of the mechanism of drug resistance, immunotherapy and epigenetic regulation have gradually been added to the treatment regimen after CDK4/6 inhibitor resistance. However, due to the difference in baseline treatment background and the heterogeneity of existing data, although more patients can benefit, there are still some patients who cannot withstand the serious adverse reactions of the treatment. Therefore, trying to explore the early benefit groups, fully screen treatment-sensitive drugs, and conduct personalized assessment according to the specific conditions of patients may bring treatment benefits to more patients.
